# Reproducibility of Air Displacement Plethysmography in Term and Preterm Infants—A Study to Enhance Body Composition Analysis in Clinical Routine

**DOI:** 10.3390/nu16121810

**Published:** 2024-06-08

**Authors:** Lennart Lücke, Christoph Fusch, Katja Knab, Stefan Schäfer, Jasper L. Zimmermann, Ursula Felderhoff-Müser, Anastasia Meis, Stephanie Lohmüller-Weiß, Adel Szakacs-Fusch, Niels Rochow

**Affiliations:** 1 Department of Pediatrics I, Neonatology, Pediatric Intensive Care, and Pediatric Neurology, University Hospital Essen, University of Duisburg-Essen, Hufelandstr. 55, 45147 Essen, Germany; luecke.lennart@gmail.com (L.L.);; 2 Research Department of Child Nutrition, University Hospital of Pediatrics and Adolescent Medicine, St. Josef-Hospital, Ruhr University Bochum, 44791 Bochum, Germany; 3 Department of Pediatrics, University Hospital Nuremberg, Paracelsus Medical University, Breslauer Str. 201, 90471 Nürnberg, Germany; christoph.fusch@klinikum-nuernberg.de (C.F.); katja.knab@klinikum-nuernberg.de (K.K.); stefan.schaefer@klinikum-nuernberg.de (S.S.);; 4 Department of Pediatrics, McMaster University, Hamilton, ON L8S 4L8, Canada; 5 DeuZWEG German Center for Growth, Development and Health Encouragement during Childhood and Youth, 10249 Berlin, Germany; 6 Department of Pediatrics, University Medicine Rostock, 18057 Rostock, Germany

**Keywords:** neonate, air-displacement plethysmography, body composition, reproducibility, lean mass, method analysis

## Abstract

The quality-initiative analysis of weekly duplicate PEAPOD^®^ body composition measurements was conducted from clinical practice (January to September 2021) on preterm and term infants without respiratory support. Statistical analysis, including regression analysis, Bland–Altman plots and cv-root-mean-square tests, was performed. A total of 188 duplicate (376 individual) measurements were collected from 119 infants (88 preterm, 31 term). The median absolute difference between duplicates was 31.5 g for fat-free mass (FFM). Linear correlation analysis showed R^2^ = 0.97 for FFM. The absolute differences in FFM and fat mass did not significantly correlate with increasing age. The %FFM differed (*p* = 0.02) across body weight groups of 1 kg < BW ≤ 2 kg (1.8%; IQR: 0.8, 3.6) and BW > 3 kg (0.9%; IQR: 0.3, 2.1). The median absolute differences were 1 g (IQR: 0.4, 3.1) for body weight and 5.6 mL (IQR: 2.1, 11.8) for body volume. Body volume estimation is charged with a constant absolute error, which is the main factor for differences between repeated body composition assessments. This error becomes more prominent in infants with lower body weights. Nevertheless, reproducibility of weekly PEAPOD testing is sufficient to monitor body compartment changes, offering a foundation for nutritional decisions in both preterm and term infants.

## 1. Introduction

Improving survival rates and early weaning from respiratory support [1,2,3] among preterm infants allows to focus on postnatal growth by optimizing enteral nutrition to achieve appropriate weight gain to ultimately improve the quality of survival. Accurate body composition data could provide key information about nutritional status in newborns and provide guidance for dietary intake. Indeed, according to the recommendations of the American Academy of Pediatrics (AAP), namely, that “… preterm infants should achieve rates of growth and composition of weight gain for a normal fetus of the same postmenstrual age and to maintain normal concentrations of blood and tissue nutrients…”, measurement of body composition should become an integral part to monitor the quality of somatic growth of preterm infants. Several methods, such as bioimpedance analysis, dual-energy X-ray absorptiometry and air displacement plethysmography (ADP), have been evaluated to assess body composition in newborns [4]. Limitations, however, for each of these methods were identified, hindering measurements in clinical practice, especially in the preterm population. Recently, routine body composition assessments using the ADP method (PEAPOD, Cosmed, Italy) were successfully introduced in a few neonatal intensive care units [5,6]. The PEAPOD, the only commercially available ADP-based body composition device for infants, was validated in multiple comparative studies; this device has shown high accuracy in term and preterm infants and is by some authors even seen as the gold standard method [7,8,9,10]. Data on the reproducibility of the test method for low percent fat mass, however, are inconclusive [11] and have not yet been assessed in a larger population of preterm infants during the first weeks of life.

Inherent in the measurement principle, ADP testing is limited to clinically stable infants without a need for respiratory support. Improvements in neonatal care and earlier weaning from continuous respiratory support currently allow ADP to be used in preterm infants with lower body weights. ADP has been validated for older infants [8,10]. However, data on preterm infants during the first month of life are scarce, raising the question of whether analysis of body composition using ADP is reliable in this vulnerable patient group.

Our group has longstanding experience in the field of neonatal body composition and has validated diverse methods, including bioelectrical impedance, skin fold thickness, dual-energy X-ray absorptiometry and ADP. Based on our previous experience from clinical ADP research at the McMaster Neonatal Intensive Care Unit (NICU), Hamilton, Ontario, we introduced weekly ADP measurements in the fall of 2020 into the clinical routine as a standard of care for extended anthropometry in our neonatal NICU [12]. After an initial implementation period of 6 months, it was decided to routinely perform measurements in duplicate to enhance the analytical precision. The present study is a quality improvement initiative to assess for the first time the reproducibility of ADP in newborns with a low fat mass and during very early life. Based on the reproducibility of the data, we aimed to establish recommendations for a reasonable time interval between follow-up measurements using ADP to reliably identify the effects of nutrition on body composition.

## 2. Materials and Methods

### 2.1. Study Design

This quality improvement initiative was performed from March until September 2021 at the neonatal intensive care unit of the Children’s Hospital at Nuremberg General Hospital, South Campus of Paracelsus Medical School Nuremberg. Using the inclusion criteria, infants were selected from our REDCap NICU database [13,14]. Anonymized data were exported and accessed from October 2021 until December 2021. All preterm and term infants without respiratory support were eligible for weekly ADP body composition assessment for extended anthropometry as an integral part of clinical routine. PEAPOD measurements were only performed in clinically stable infants who fulfilled the following inclusion criteria: no respiratory support, FiO_2_ of 0.21, no episodes of significant desaturation (episodes of SaO_2_ < 85% for >15 s) or bradycardia (<80 bpm) requiring stimulation within the last 48 h. The data were extracted from the PEAPOD database and anonymized for analysis.

Prior to the study, the measurement protocol was approved by our institutional review board (#SZ_D_028.21-IX-1). According to German professional regulations for physicians, the study did not require additional Ethics Committee approval because it was considered to be a quality improvement initiative, with all prior data being available on a routine basis and analyzed in an anonymized way, which was also reported according to the Standards for Quality Improvement Reporting Excellence (SQUIRE 2.0) [15]. For all procedures applied to our NICU patients, we informed their parents or legal guardians orally and in writing about our standards of care and routine procedures, their indications, their nature, risks and benefits, and this information was documented by the physician’s and parents’ signatures. The use of extended anthropometry by ADP is included herein.

### 2.2. Nutrition

All infants were fed according to our local clinical guidelines [16]. In general, infants with a birth weight < 1000 g or born at a gestational age < 28 + 0/7 weeks were fed an exclusively human milk diet including the human milk-based fortifier and ready-to-feed milk (Humavant, Prolacta Bioscience Inc., Groot-Bijgaarden, Belgium) used for the first 4 weeks after reaching full enteral feeding (150 mL/kg/d). Preterm infants with a gestational age of 28 + 1/7 to 33 + 6/7 weeks received mother’s own milk (MOM) that was target bovine fortified (Aptamil FMS, Danone GmbH, Frankfurt, Germany) or preterm formula at 80 kcal/100 mL (Aptamil Prematil, Danone GmbH, Frankfurt, Germany). Breastmilk analysis for target fortification was performed twice per week, and the macronutrient content was adjusted using modulars to reach the ESPGHAN recommendations [12,17,18]. Infants born at a gestational age of 34 + 0/7 to 36 + 6/7 weeks received standard fortified MOM (Aptamil FMS, Danone GmbH, Frankfurt Germany) or preterm formula at 73 kcal/100 mL (Aptamil PDF, Danone GmbH, Frankfurt, Germany). Term-born infants (≥37 + 0/7) were fed MOM or term formula (Aptamil Pronutra Pre, Danone GmbH, Frankfurt, Germany).

### 2.3. Testing Routine

Weekly routine body composition measurements were conducted at a predefined time window between 8.30 AM and 11.00 AM. Duplicate measurements were performed to evaluate the reproducibility by analyzing the difference between these two measurements. The measurements were performed in the following sequence: (i) body length using a length board, (ii) head circumference using nonstretchable tape, (iii) first PEAPOD measurement (body weight, body volume), and (iv) second PEAPOD measurement (body weight, body volume). A single PEAPOD measurement takes approximately 2–3 min. Duplicate measurements were performed consecutively within a period of 10 min.

Body weight was measured to a resolution of 0.1 g using the digital scale integrated within the PEAPOD. The resolution of the length board (Infantometer Seca 416, Hamburg, Germany) and nonstretchable tape was 1 mm. The measurements were performed by one device operator assisted by trained research nurses in one designated room. The room temperature was maintained at 26 °C. The PEAPOD device was not moved throughout the study period.

### 2.4. Body Composition and Anthropometric Measurement

Body composition (fat mass, percent fat mass, fat-free mass) assessments were performed using PEAPOD (Cosmed, Rome, Italy) based on the principle of ADP [19]. ADP relies on a two-compartment model that divides the body into fat mass and fat-free mass. Fat mass and fat-free mass are derived from body density. Body density was calculated as the ratio of body weight to body volume. Body weight was measured using the inbuilt PEAPOD scale. Body volume was obtained as the difference in the total volume of the empty measurement chamber minus the volume of compressible air after the baby was placed inside. 

### 2.5. Methodology of Air Displacement Plethysmography

ADP uses a two-compartment model which divides the body into fat (FM) and fat-free mass (FFM). Compartment sizes are calculated from a subject’s body density assuming known values for the densities of FM and FFM. Body density is calculated from body volume and body weight with body weight being measured by the inbuilt scale. Body volume is measured as residual volume of compressible air in the measurement chamber with and without the subject in place, thereby applying the PV = nRT relationship of the ideal gas law (P: pressure; V: volume; T: temperature; n: number of moles; R universal gas constant) of compressible gases. Body volume measurement thereby relies on two main assumptions:

Density of fat mass is assumed to be constant (0.9007 kg/L), whereas density of fat-free mass depends on gestational age. Reference data for fat mass and fat-free mass were used from Butte et al. and Fomon et al. [20,21]. 

Intrathoracic air and air in the proximity of skin is overestimated by 40% because of isothermal characteristics. To correct for temperature or humidity shifts, the correction algorithm requires to calculate the functional residual capacity of the lung as well as the amount of air volume near the body surface. Equations for both parameters therefore incorporate natural constants, body weight and length [20]. 

Based on these assumptions, body composition is calculated by the following equation:

(1) $$\frac{1}{D_{B}}=\frac{F}{D_{F}}+\frac{FFM}{D_{FFM}}$$

(2) $$\%\mathrm{fat}=\left[\frac{D_{F}D_{FFM}}{D_{B}\left(D_{FFM}-D_{F}\right)}-\frac{D_{F}}{D_{FFM}-D_{F}}\right]\times 100\backslash \%$$

Algorithm used by ADP to calculate Body composition results (D_B_ = KG/V (body weight/body volume, determined by PEAPOD); D_F_ = density of fat (known from literature); D_FFM_ = density of fat-free mass (known from literature); F = fat mass (g); FFM = fat mass (g); %fat = percentage fat mass 

A more profound description of measurement procedure, technical information and physical mechanics of the PEAPOD device can be found in the methodical articles by Yao et al. and Urlando et al. [19,20].

### 2.6. Methodology of the Theoretical Estimation of Error Propagation Due to Error in Duplicate Body Volume Measurements

How the error occurring during body volume assessment translates to the precision of body composition analysis was studied using a theoretical model. Error propagation in duplicate body volume measurements was estimated using a theoretical model. For this purpose, a body volume test-retest difference of 10 mL was applied to two hypothetical infants of the same age (1 month) but with different body weights (1 kg and 2 kg). The difference of 10 mL was chosen to be close to the expected differences of body volume measurement (5.6 mL; 3rd quartile: 11.8 mL) as reported in our cohort. According to the principles of ADP, both infants have identical densities of fat-free mass (FFM) and fat mass (FM) (the age-adjusted density of FFM is 1.064 kg/L, and the constant density of FM is 0.9007 kg/L) [20,21,22]. The calculation of theoretical body composition was then performed using a previously published algorithm (see Section 2.5. Methodology of air displacement plethysmography).

### 2.7. Data Analysis

Descriptive statistics were performed for body composition and anthropometric data.

Differences between duplicate measurements were assessed by absolute difference, Bland–Altman plots [23] and linear regression analysis. The coefficient of determination (R^2^), slope and intercept were analyzed to quantify the differences. Repeated measurements were considered nonidentical because of potential changes in body weight due to passing through urine or stool as well as temperature differences at the body surface.

Differences in duplicate measurements were also compared between four subgroups for postnatal age (PNA) and three subgroups for body weight (BW). PNA ≤ 1, 1 < PNA ≤ 2, 2 < PNA ≤ 3 and 3 < PNA ≤ 4 months were defined as the PNA 1, PNA 2, PNA 3 and PNA 4 groups, respectively, and 1 < BW ≤ 2, 2 < BW ≤ 3 and 3 < BW < 4 kg were defined as the BW 1, BW 2 and BW 3 groups, respectively. Differences between subgroups for fat-free mass and fat mass percentage were analyzed by the Mann–Whitney U-test and ANOVA. The reproducibility of the fat-free mass and fat mass percentage was characterized by the standard deviation, median and interquartile range (IQR). The experimental reproducibility of the fat-free mass and fat mass percentage was analyzed using the root-mean-square coefficient of variation (CV-RMS) for the total population and the two groups of PNA (<1 and ≥1 month; 1 month defined as 31 days), as follows: CV-RMS = √Σ(CV2/n) [11]. CV-RMS was utilized to precisely identify outlier measurements, ensuring a higher level of precision in the detection of deviation between duplicate measurements and allowing comparison with published reference studies [11]. The impact of potential errors from body length measurements was assessed in a subgroup of n = 5 infants for differences in body length of (−2 cm, −1 cm, +1 cm, +2 cm), thereby keeping body weight constant. Statistical analysis was performed using SPSS (Statistics for Windows, Version 28.0, released 2021, IBM Corp, Armonk, NY, US). Data management was performed with Microsoft Excel^®^ (Microsoft 365, MSO Version, 2022, Redmond, WA, US). Graphic design was performed using GraphPad Prism (version 9 for Windows, GraphPad Software, San Diego, CA, US).

## 3. Results

One hundred nineteen infants were included (n = 88 preterm infants, n = 31 term infants). A total of n = 188 ADP measurements were performed in duplicate (total of n = 376 individual measurements) (Table 1). A total of 155 duplicate measurements were performed for 88 preterm infants, and 33 duplicates were performed for 31 term infants.

### 3.1. Differences in Fat-Free Mass and Fat Mass

**Figure 1:**
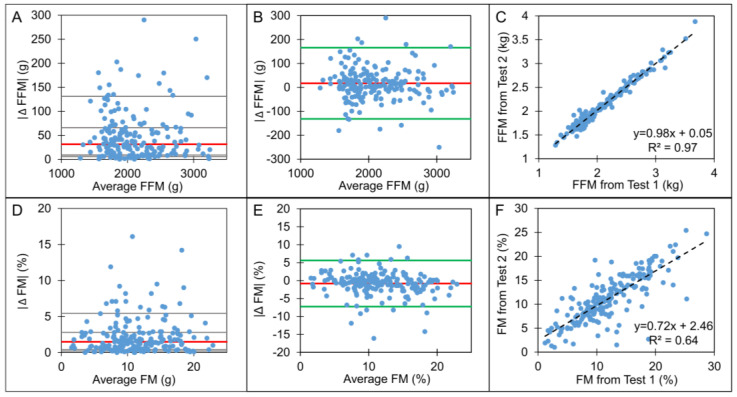
(**A**–**F**): **Comparison of fat-free mass (FFM) and %fat mass (%FM) from duplicate tests (n = 188).** Panels (**A**,**D**): absolute differences |Δ| of FFM and %FM per average FFM and %FM. (Red line: median difference; gray lines: 10th, 25th, 75th, 90th percentiles). Panels (**B**,**E**): Bland–Altman plots of FFM and %FM (difference Δ defined as Test2–Test1). (Red line: mean difference, green line: ±2 standard deviations). Panels (**C**,**F**): scatter plots of FFM and %FM (black dashed line: linear regression line).

The median absolute differences between duplicate measures of fat-free mass and percentage fat mass were 31.5 g and 1.5%, respectively (Figure 1A,D). This difference comprises 1.5% of the fat-free mass and 13% of the fat mass compartment. Bland–Altman analysis for duplicate fat-free mass showed strong agreement between tests, with a mean bias of 17 g being not significantly different from zero (95% CI: −131 g, 165 g, Figure 1B). The correlation analysis showed a small deviation from the line of identity and a high agreement (R^2^ = 0.97) with linear regression (Figure 1C). Bland–Altman analysis for fat mass percentage showed limited agreement between tests, with a mean bias of 0.8% (95% CI: −7.2%, 5.6%, Figure 1E). The results of the linear regression analysis differed from the line of identity (R^2^ = 0.64) (Figure 1F). 

A subgroup analysis stratified by age at measurement revealed a lower mean %fat mass in infants less than one month old than in infants more than or equal to one month of age (10.1% and 16.9%, respectively). The root mean square-CV, as a measure of the reproducibility of fat-free and %fat mass, differed between the two age groups, showing greater reproducibility in older infants. For infants younger than one month and equal to or older than one month of age, the root mean square CVs were 19.9% and 7.1% for %fat mass and 2.1% and 1.4% for fat-free mass, respectively (Table 2).

**Figure 2:**
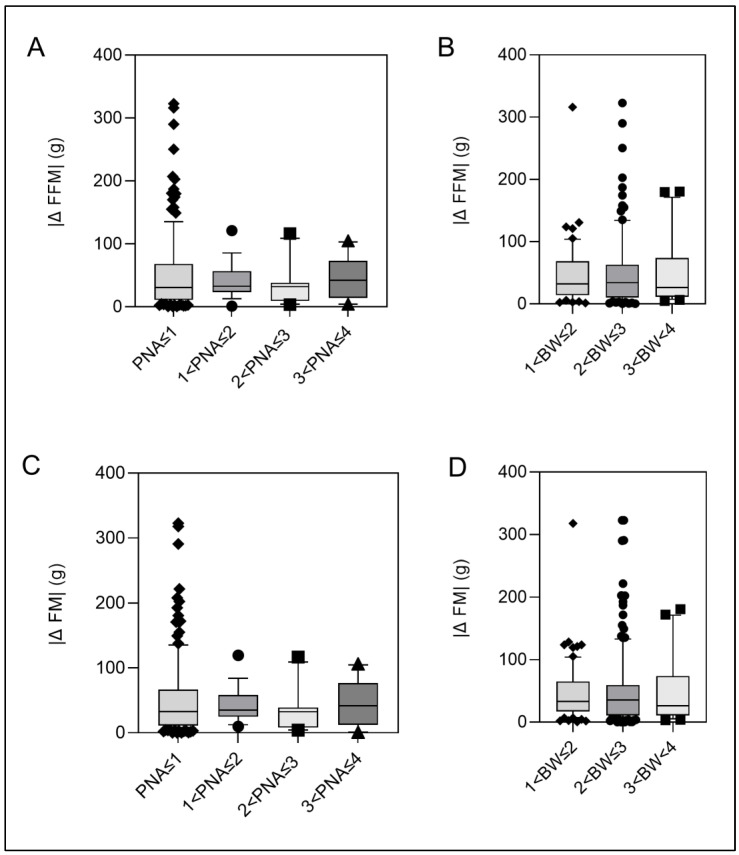
(**A**–**D**)**: Absolute differences |Δ| of duplicate measurements of fat-free mass (FFM) and fat mass (FM).** Box-whisker plots characterize median differences using the 10th, 25th, 50th, 75th and 90th percentiles. Black dots: data points < 10th and >90th percentile. Panels (**A**,**C**) are stratified by postnatal age. Panels (**B**,**D**) were stratified for body weight.

Linear regression analysis of potential errors in fat mass estimation for differences in body length (−2 cm, −1 cm, +1 cm, +2 cm) indicated that for each centimeter of body length, fat-free mass estimation will increase by 5 g. The absolute value of the difference |Δ| between duplicate measurements of fat mass and fat-free mass estimation did not vary significantly between the different age or weight groups (Figure 2).

**Figure 3:**
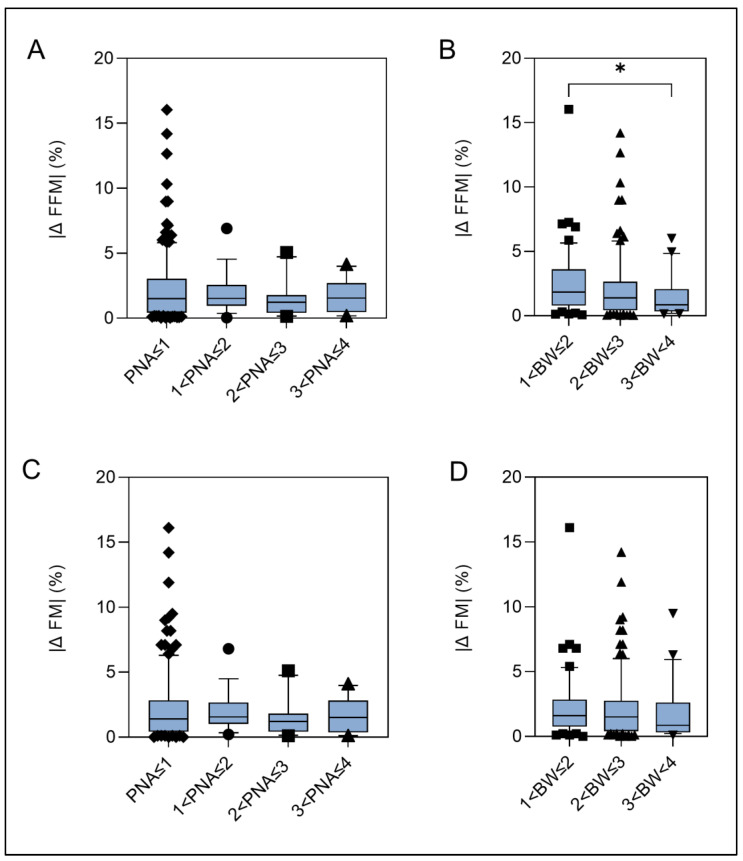
(**A**–**D**)**: Absolute differences |Δ| of duplicate measurements of %fat-free mass (%FFM) and %fat mass (%FM).** Box plots characterize median differences using the 10th, 50th and 90th percentiles. Black dots: Deviating data points < 10th and >90th percentile. Panels (**A**,**C**) are stratified by postnatal age. Panels (**B**,**D**) were stratified for body weight. * represents statistical significance at *p* < 0.05.

To assess the comparability between individuals, postnatal age and body weight were stratified and analyzed for intergroup differences in %fat-free mass and %fat mass (per kg body weight). The absolute differences |x| of duplicate measurements of %fat-free mass and %fat mass significantly decreased with increasing body weight and were 1.8% (IQR: 0.8, 3.6) for infants in the BW 1 group and 0.9% (IQR: 0.3, 2.1) for infants in the BW 3 group (*p* = 0.02). In the PNA 1 group and PNA 4 group, the differences in fat-free mass were minimal at 1.5% (IQR: 0.4, 3.1) and 1.6% (IQR: 0.5, 2.7), respectively. For %fat mass, the differences were 1.6% (IQR: 0.8, 2.9) and 0.9% (IQR: 0.3, 2.6) for the BW 1 group and BW 3 group, respectively, as well as 1.4% (IQR: 0.4, 2.9) and 1.5% (IQR: 0.4, 2.8) for the PNA 1 and PNA 4 groups, respectively (Figure 3C,D). ANOVA of %FFM and %FM did not show a significant difference between the PNA or BW groups. However, for %FFM, the differences between the BW groups were close to the level of significance (*p* = 0.06).

### 3.2. Differences in Body Weight and Body Volume Estimation 

**Figure 4:**
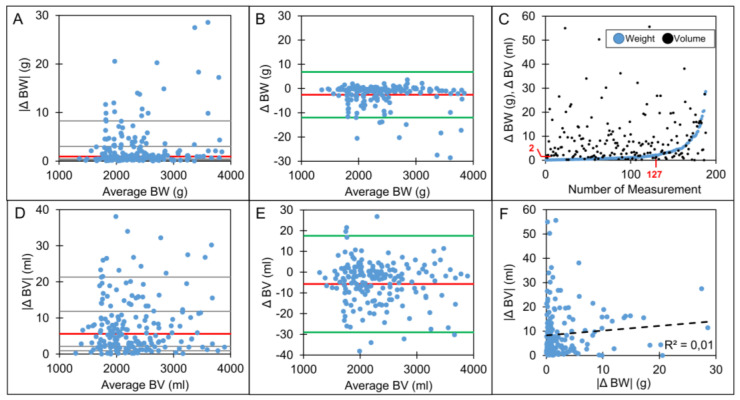
(**A**–**F**)**: Test**–**retest differences in body volume (BV) and body weight (BW) (n = 188).** Panels (**A**,**D**): Median absolute differences |Δ| of BW and BV per mean BW and BV (Red line: median difference, gray lines: 10th, 25th, 75th, 90th percentiles). Panels (**B**,**E**): Bland–Altman plots of BV and BW: difference Δ defined as test 2-test 1 (red line: mean difference, green line: ± 2 standard deviations). Panel (**C**): scatter plot of ΔBV and ΔBW sorted in increasing order for ΔW (Red line: cutoff at a ΔBW of 2 g at test number 127 of 188). Panel (**F**): scatter plot of ΔBV and ΔBW (black dashed line: linear regression line).

Test-retest analysis of body weight and body volume for duplicate measurements revealed high reproducibility. For both, linear regression of duplicate measurements showed high agreement (R^2^ = 0.99). The median absolute difference in estimated body weight was 1 g (1st quartile: 0.4 g, 3rd quartile: 3.1 g, Figure 4A), and the median body volume was 5.6 mL (2.1 mL, 11.8 mL, Figure 4D). Bland–Altman analysis revealed a constant difference in both body weight and body volume over the full range of body weight measurements from 1.5 to 4 kg (Figure 4B,E). The absolute difference in body weight was ≤2 g in two-thirds (127 out of 188) of the measurements (Figure 4C). Linear regression revealed no associations between absolute differences in body weight and volume (R^2^ = 0.01) (Figure 4F) or between differences in body weight and differences in fat mass percentage (R^2^ < 0.01).

### 3.3. Theoretical Estimation of Error Propagation Due to Error in Duplicate Body Volume Measurements

Infant A, with a body weight of 1000 g, had 35.3% fat mass when the body volume was 1000 mL but 29.4% fat mass when the BV was 990 mL. The absolute difference in FM between both measurements was 62 g.

**Figure 5:**
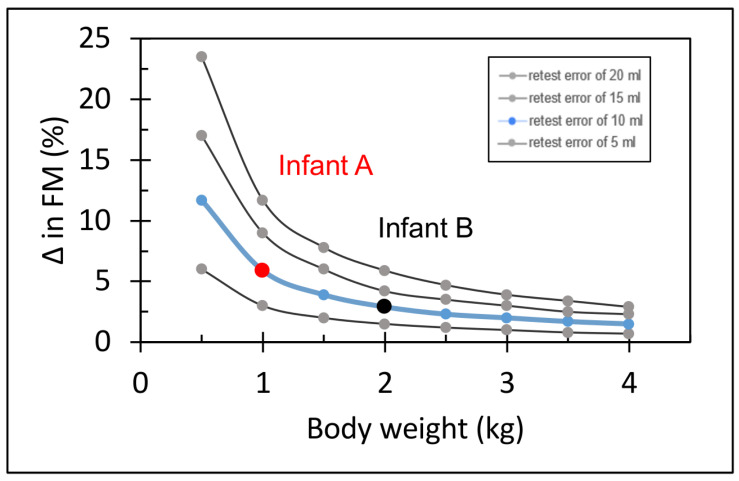
Theoretical model analyzing the test-retest error of %fat mass (FM) estimation for different body volume errors. The *y*-axis represents the absolute %FM difference.

Infant B, however, with a body weight of 2000 g, had a 35.3% fat mass when the body volume was 2000 mL but had a 32.4% fat mass body volume when the body volume was 1990 mL. The absolute difference for FM between both measurements is 62 g. An identical error of body volume measurement (10 mL) results in an error of %fat mass estimation of 6% (35.3–29.4 %FM) for infant A and 3% (35.3–32.4 %FM) in infant B. Detailed error propagation for different retest error values (5, 10, 15 and 20 mL) and for different body weights is presented in Figure 5.

## 4. Discussion

To our knowledge, this is the largest study in preterm and term infants within the first weeks of life to investigate the reproducibility of ADP measurements of neonatal body composition between tests and retests. Overall reproducibility for our cohort was high for fat-free mass estimation but lower for fat mass. The |Δ| between duplicate measurements for FM and FFM was constant across the age and body weight ranges assessed. We found a significant decrease in the relative errors of %FFM with increasing body weight. Hence, relative differences are greater in infants tested shortly after birth or those with a low body weight. A constant error of absolute size for body volume estimation was identified as the major factor influencing test-retest reliability. Based on the confidence intervals characterizing the precision of ADP, we propose minimum intervals for repeated body composition measurements to reliably assess changes in body compartment sizes between two time points with sufficient statistical power.

In our cohort, fat mass was the smaller compartment of total body mass, up to a factor of eight when compared to fat-free mass. Hence, a small imprecision of compartment size estimation has an 8-fold greater effect on fat mass percentage when compared to fat-free mass compartment (mean of 88.5% of the body mass). As a result, fat mass percentage is the parameter most affected by errors in ADP tests and could preferably be used for reproducibility and accuracy analysis.

To study where differences between duplicate body composition measurements originate, we stratified the analysis by postnatal age and weight group. The absolute differences between measurements were identical for both fat and fat-free mass and only marginally changed with increasing age or body weight. This observation can be explained by the fact that the PEAPOD is based on the two-compartment model. Accordingly, the errors of FFM analysis are reciprocal and mirror those of FM at equal absolute amounts.

At the age of one month and older, our results showed reproducibility (mean |Δ| FM: 1.8 ± 1.5%; median |Δ| FM: 1.4, IQR: (0.5, 2.5)) and were comparable with published data. Roggero et al. showed 95% limits of agreement of −1.9 to 2.7% fat mass percentage (n = 70 preterm, n = 9 term infants) [8]. Ellis et al. (N = 31 newborn infants) and Ma. et al. (n = 36 term infants) reported slightly greater reproducibility for fat mass percentage, with mean differences of 0.41 ± 1.3% and 0.16 ± 1.44%, respectively [7,9]. However, none of the three studies analyzed the absolute differences in |Δ| between measurements. We believe that the absolute differences must be analyzed due to the random nature of the error.

However, in the group younger than one month, our data showed decreased reproducibility (mean Δ%FM: 2.3 ± 2.8%, RMS-CV: 19.9%). Similar findings were reported by Frondras-Chauty et al. with duplicate ADP measurements in piglets aged 2–21 days (RMS-CV: 17.9%) [11].

### 4.1. Factors Contributing to Differences between Duplicate Assessments

However, the differences in ADP between duplicate ADP body composition assessments may be affected by external and internal factors.

The following external factors reflect the changing conditions of the subjects or of the environment during testing:

- Changes in ambient temperature: The ADP uses the pressure–volume–temperature (PVT-R) relationship to measure the compressible gas volume in the measurement chamber. Hence, the ADP depends on the ambient temperature, and the accuracy and precision depend on the stability of the ADP. Therefore, all measurements were performed at identical locations—a room dedicated only for PEAPOD measurements near the neonatal intensive care unit. On test days, the room was heated to a constant temperature of 26 °C to prevent heat loss in preterm infants. In this way, the impacts of temperature changes on the accuracy and precision of the measurements were minimized. Changes in skin temperature or hair volume: the air layer adjacent to the skin is physically different in terms of temperature and humidity, and the PVT-R may be different. The ADP corrects for this effect, thereby making some assumptions. However, the actual study protocol requires the ADP to be recalibrated between the first and second measurements. This process takes approximately 3 min, during which the baby is not allowed to remain in the measurement chamber. The infants were held in nurses’ arms or placed under a heat lamp and then returned to the incubator. The difference in skin temperature between the first and second measurements could have potentially led to isothermal effects in the proximity of the skin, thus affecting the volume calculation. For similar reasons, the infants’ hair was oiled before every first body composition assessment to avoid errors in the assessment of body volume. Oiling was not repeated for the second test, which may have led to a difference in body volume estimation. The impact of these two factors could not be retrospectively quantified.
- Changes in body weight due to passing through urine or stool: During the testing process, we observed urination and defecation in a small number of infants. The amount of urine or defecation was not quantified but could have caused errors in body weight and volume assessment. To prevent this error, two consecutive measurements without passing through urine or stool should have been performed. If passing urine or stool was responsible for a significant error between repeated body weight measurements, a comparable error of body volume and body composition estimates would have to be expected. In contrast, linear regression analyses revealed no statistically significant correlation between the first and second measurements of body weight and body volume (R^2^ = 0.01) or between differences in body weight and fat mass (R^2^ < 0.01). These results suggest that potential weight and volume differences due to passing through urine or stool do not seem to be the main causes of the errors in volume and fat mass estimation in duplicate testing.
- Inaccuracies of body length measurement: Our calculation shows that, per cm increase in body length, fat-free mass will increase by 5 g, and fat mass will decrease by the same amount. With an imprecision of ±0.5–1.5 cm, body length measurements are typically charged with this error, which might become clinically significant in subjects with lower body weight and may be on the same order of magnitude as body volume imprecision [24]. These findings clearly emphasize the importance of accurate body length measurements in clinical practice. Using the mean/median or, alternatively, the highest one of repeated measures should be considered. This potential error, however, does not apply to our study setting since the testing sequence of duplicate measurements contained a single body length measurement only.

The following internal factors may cause inaccuracies related to the ADP method:

- Methodological limitations in the estimation of thorax volume or body surface area: Due to isothermal properties, the ADP tends to overestimate compressible thoracic volume and air volume in proximity to the skin. PEAPOD uses an equation to adjust for this potential overestimation. The equation includes physical constants, body weight and length. Test-retest differences in body length and weight could lead to imprecision in body composition measurements.
- Methodological errors in the estimation of body volume and body weight: Body volume and body weight are the main variables measured and are used by the PEAPOD algorithm to estimate body composition. The reproducibility was high for both parameters. The mean differences in body volume were significantly greater than the mean differences in body weight and were constant over the whole range of body weights measured. Hence, a constant error in body volume can potentially explain why the relative error of body composition estimation decreases in older infants with greater body weight. This hypothesis was subsequently tested using a theoretical model to calculate the error in body composition estimation introduced by test-retest differences in body volume estimation (10 mL). The results from this theoretical model match the observations of our reproducibility analysis (Figure 5). Future studies should investigate whether a correction formula for the body volume assessments could improve the reproducibility of PEAPOD. Validation of this formula would have to be analyzed by correlating results against an additional reference method for body composition assessments.
- We conclude that a small but constant error in body volume assessment will cause an error in the calculation of body composition and explain the lower reproducibility in infants with low body weight.

These observations are clinically relevant because they may provide guidance when performing ADP body composition analysis, such as which time interval (days or weeks) would be reasonable for ordering follow-up measurements to identify the effects of nutrition on body compartment growth. Our data show a median difference (+3rd quartile) of ~40 g/kg in fat-free mass estimations in infants with a body weight less than 3 kg (Figure 3). In this group, the fat-free component comprises approximately 90% of the total body weight (Table 2), and the weight gain velocity is expected to be ~15 g/kg/d [25], leading to a fat-free mass gain velocity of 13.5 g/kg/d. At this mean growth velocity, a reasonable margin of 2 standard deviations (~100 g/kg for fat-free mass) would be ensured at intervals for follow-up body composition assessments of 7–8 days, e.g., once per week. 

### 4.2. Strength and Limitations

A limitation of this study is that duplicate PEAPOD measurements only allow analysis of reproducibility. The lack of a reference method for body composition did not allow analysis of accuracy. A further limitation of this study is that data on primary parameters such as measured pressure differences and changes in temperature are not available to the user. The device only provides body volume data directly calculated from measured pressure–temperature differences, which limits the identification of measurement errors. Another methodological drawback of our study was that the quantification of urine and defecation during the testing process could have been useful in understanding the value of this error.

A strength of our study is that PEAPOD tests were integrated into routine clinical practice, and the study was performed under real-life conditions. All testing was performed by one device operator only, reducing measurement bias. Another strength of this study is that only healthy infants without respiratory support were tested in clinical practice, providing a large, homogeneous cohort of both preterm and term infants with a total of 376 measurements.

## 5. Conclusions

In conclusion, our study identified an error of constant size for body volume estimation, which leads to an imprecision of duplicate body composition assessments by ADP. This error is more prominent in infants with low body weight (<3 kg). However, at weekly testing intervals, the reproducibility of PEAPOD appears to be sufficient to visualize body composition growth, enabling nutritional intervention in both preterm and term infants.

## Figures and Tables

**Table 1:** Infant characteristics at first body composition test day (GA—Gestational age, PMA—postmenstrual age, PNA—postnatal age).

	Preterm	Term	Total
Number of infants Sex (M/F) GA (weeks)	88	31	119
	50/38	19/12	69/50
	33.0 ± 3.0 (24–36.7)	39.2 ± 1.5 (37–41.8)	34.6 ± 3.8 (24–41.8)
Body weight at first test (kg) Length at first test (cm)	2.2 ± 0.5	3.0 ± 0.7	2.4 ± 0.6
	42 ± 2.4	49.1 ± 3.0	45.7 ± 2.9
PMA at first test (weeks) PNA at first test (weeks)	35.9 ± 1.8	39.9 ± 1.5	36.6 ± 2.3
	3.6 ± 4.1	0.8 ± 0.9	3.1 ± 3.9

**Table 2:** Patient characteristics at test day and differences in mean fat-free mass and fat mass percentage and CV-RMS from duplicate tests per group of postnatal age (PNA). CV-RMS: Root-mean-square coefficient of variation (utilized to precisely identify outlier measurements) = √ 100 × SD/mean [%], FFM: fat-free mass [g], %FM: %fat mass [%], PMA: postmenstrual age [wks], |Δ|: absolute differences.

Postnatal Age (PNA)	<1 Month	≥1 Month
Patient characteristics at measurement		
Number of duplicate tests	149	39
PMA at test day (weeks)	36.4 ± 2.2	37.3 ± 2.6
Week of life	1.4 ± 1.0	9.7 ± 4.1
	Test results	
FFM mean (kg)	2.1 ± 0.5	2.1 ± 0.4
FM mean (%)	10.1 ± 4	16.9 ± 4.3
	Difference	
Median |Δ| FFM (g)	33.2, IQR: (11.4, 69.9)	29.3, IQR: (18.0, 56.9)
Median |Δ| FM (%)	1.5, IQR: (0.4, 2.9)	1.4, IQR: (0.5, 2.5)
Mean (±SD) |Δ| FFM (g)	53 ± 62	43 ± 35
Mean (±SD) |Δ| FM (%)	2.3 ± 2.8	1.8 ± 1.5
FFM CV-RMS	2.1	1.4
%FM CV-RMS	19.9	7.1

## Data Availability

The original contributions presented in the study are included in the article, further inquiries can be directed to the corresponding author.
